# Functional Magnetic Resonance Spectroscopy: The “New” MRS for Cognitive Neuroscience and Psychiatry Research

**DOI:** 10.3389/fpsyt.2018.00076

**Published:** 2018-03-12

**Authors:** Jeffrey A. Stanley, Naftali Raz

**Affiliations:** ^1^Department of Psychiatry and Behavioral Neurosciences, School of Medicine, Wayne State University, Detroit, MI, United States; ^2^Department of Psychology, Wayne State University, Detroit, MI, United States; ^3^Institute of Gerontology, Wayne State University, Detroit, MI, United States; ^4^Center for Lifespan Psychology, Max Planck Institute for Human Development, Berlin, Germany

**Keywords:** MRI, ^1^H MRS, glutamate, cognition, plasticity, schizophrenia, aging

## Abstract

Proton magnetic resonance spectroscopy (^1^H MRS) is a well-established technique for quantifying the brain regional biochemistry *in vivo*. In most studies, however, the ^1^H MRS is acquired during rest with little to no constraint on behavior. Measured metabolite levels, therefore, reflect steady-state concentrations whose associations with behavior and cognition are unclear. With the recent advances in MR technology—higher-field MR systems, robust acquisition techniques and sophisticated quantification methods—^1^H MRS is now experiencing a resurgence. It is sensitive to task-related and pathology-relevant regional dynamic changes in neurotransmitters, including the most ubiquitous among them, glutamate. Moreover, high temporal resolution approaches allow tracking glutamate modulations at a time scale of under a minute during perceptual, motor, and cognitive tasks. The observed task-related changes in brain glutamate are consistent with new metabolic steady states reflecting the neural output driven by shifts in the local excitatory and inhibitory balance on local circuits. Unlike blood oxygen level differences-base functional MRI, this form of *in vivo* MRS, also known as functional MRS (^1^H fMRS), yields a more direct measure of behaviorally relevant neural activity and is considerably less sensitive to vascular changes. ^1^H fMRS enables noninvasive investigations of task-related glutamate changes that are relevant to normal and impaired cognitive performance, and psychiatric disorders. By targeting brain glutamate, this approach taps into putative neural correlates of synaptic plasticity. This review provides a concise survey of recent technological advancements that lay the foundation for the successful use of ^1^H fMRS in cognitive neuroscience and neuropsychiatry, including a review of seminal ^1^H fMRS studies, and the discussion of biological significance of task-related changes in glutamate modulation. We conclude with a discussion of the promises, limitations, and outstanding challenges of this new tool in the armamentarium of cognitive neuroscience and psychiatry research.

## Introduction

Understanding of human behavior and cognition as products of their neural substrates depends on elucidation of the neural foundations of information processing. With the brain neurons comprising only about 10% of the gray matter bulk (1), allocating the lion share of brain energy supply to neurotransmission (2) suggests that deciphering the relationships between neurotransmitter dynamics and cognitive operations is key to success of that enterprise. Most (up to 80%) of cortical and hippocampal neurons are excitatory with glutamate as their dominant neurotransmitter, while the remaining 20% are inhibitory and have γ-aminobutyric acid (GABA) as their principal neurotransmitter (3). Therefore, understanding the dynamics of these neurotransmitter’s release during cognitive operations is particularly important for elucidating the mechanisms of normal and abnormal behavior. Notably, cortical glutamatergic and GABAergic neurons do not act as separate excitatory and inhibitory entities but are highly integrated into neural ensembles within local and long-range circuits, in which the “balanced” excitatory and inhibitory (E/I) synaptic drive serves as the functional basis of coherent networks (4–7).

**Figure 1:**
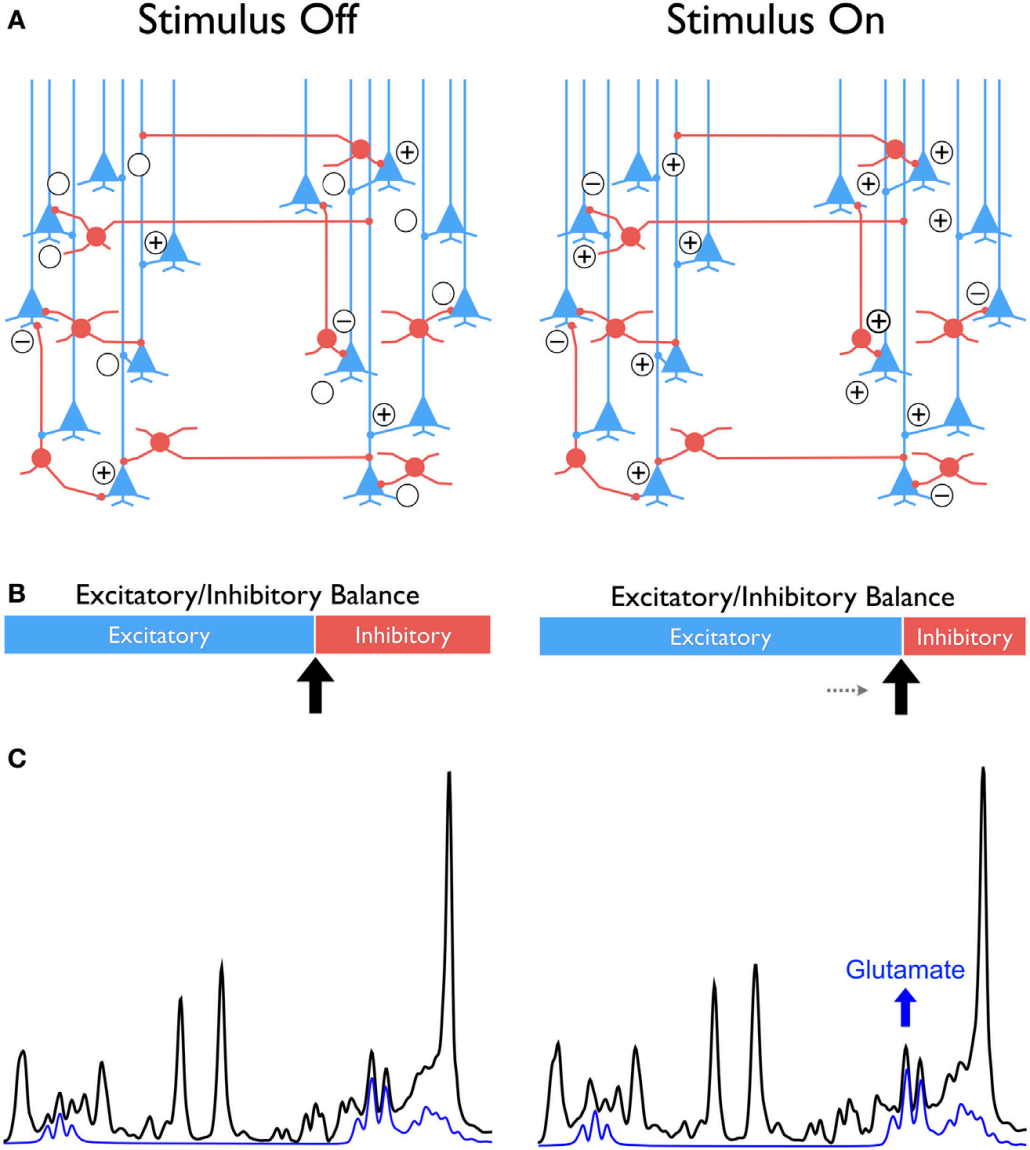
Conceptual framework comparing the “balanced” excitatory and inhibitory (E/I) synaptic drive at stimulus-free and stimulus-dependent conditions in cortex with glutamatergic pyramidal neurons in blue and GABAergic interneurons in red **(A)**. The difference between conditions is conceptualized as a shift toward greater excitability at stimulus onset compared to a no-stimulus condition that is represented as sliding bars with excitatory in blue and inhibition in red **(B)**. This shift leads to a new metabolic steady state reflected in the increased glutamate as illustrated in the individual signal in blue extracted from the ^1^H MRS spectrum shown in black **(C)**. The “+” and “−” symbols signify the excitatory and inhibitory synaptic activity, respectively.

In the cortex, sensory input, motor output as well as perceptual, and cognitive activity evoke temporally correlated excitation and inhibition at the synapses, thus shifting the dynamic equilibrium of E/I toward a (wide) range of excitation–inhibition patterns, as illustrated in Figure 1. These temporal fluctuations in E/I equilibrium eventually give rise to plasticity and synaptic reorganization by driving long-term potentiation and long-term depression, which are viewed as the neurophysiological bases of memory [see Tatti et al. (7) for a recent review]. Because of strong evidence implicating the glutamatergic and GABAergic neurotransmission in psychiatric disorders (8–10), and cognitive aging (11), it is plausible that a dysfunction in the ability to modulate E/I equilibrium of local circuits would affect function within broader networks in which complex cognition is implemented. Impairment of glutamatergic and GABAergic plasticity may underpin the development of symptomatology that characterizes psychiatric disorders (7) and age-related cognitive dysfunction (12).

Whereas in animal models, a wide range of invasive methods of gauging glutamatergic and GABAergic activity is available, in humans, the opportunities are very limited. To date, the most popular approach to studying brain correlates and neural mechanisms of cognition *in vivo* harnesses blood oxygen level differences (BOLD) effect in an imaging paradigm known as functional MRI (fMRI). Although fMRI has good temporal and spatial properties, the BOLD signal is, however, an indirect measure of the neuronal response to stimuli. In addition, the BOLD signal cannot differentiate between inhibitory or excitatory neural activity. Moreover, the BOLD signal is influenced by major determinants of vascular tone such as dopamine (5) that depends on age (13) and are altered in psychiatric conditions (14). Given the role played by glutamate and GABA to shifting the E/I balance in cortical information processing, it is critically important to develop more specific means of *in vivo* evaluation of glutamatergic and GABAergic systems in intact, behaving humans. Such noninvasive approach to assessing regional brain concentration of these important neurotransmitters is indeed available. In the neuroimaging literature, magnetic resonance spectroscopy (MRS) is typically described as the only noninvasive technique that can reliably quantify *in vivo* concentration levels of key metabolites, including glutamate and γ-aminobutyric acid (GABA) (15).

^1^H MRS, with its ability to measure glutamate and GABA levels *in vivo* in localized cortical and subcortical areas, is well suited for testing hypotheses posited in the conceptual framework that emphasizes temporal dynamics of the E/I equilibrium (Figure 1). Unfortunately, the dynamic aspect of glutamate (and GABA) activity is lost in the majority of the extant ^1^H MRS studies that are limited to measuring static neurotransmitter levels under “pseudo-” rest condition. In a typical ^1^H MRS experiment, the data are acquired without any specific instructions or behavioral constraints aside from asking the participants to relax and keeping the head still during acquisition. Thus, the measured neurotransmitter levels are static and integrated over a time window spanning several minutes. This coarse temporal resolution and static task-free neurotransmitter assessment preclude the interpretation of findings with respect to neural correlates of synaptic plasticity. Although the ^1^H fMRS literature is sparse, evidence shows surprising sensitivity in detecting dynamic changes in the magnitude and direction of task-related changes in glutamate and/or GABA steady-state levels in functionally relevant brain areas (Table 1). This ability to capture the temporal dynamics of glutamate and GABA *in vivo*, point at the emerging role of ^1^H fMRS as the “new” ^1^H MRS, with potentially exciting contributions to the understanding of neural mechanisms relevant to cognitive neuroscience and psychiatry research.

**Table 1:** Description of ^1^H fMRS studies reporting task-related changes in glutamate.

Study	Sample size	Acquisition protocol	Task	Results	Comments
**Visual stimuli—visual cortex**					
Mangia et al. (41)	12 adults	– 7 T – STEAM TE = 6ms – Midline visual cortex – 2 cm × 2.2 cm × 2 cm	– Radial red/black checkerboard covering the entire visual field (8 Hz) – Two protocols: (1) 2 short 5.3 min blocks interspersed by rest epochs and (2) 1 long 10.6 min block interspersed by rest epochs	– Increased glutamate (3%) during checkerboard vs rest	– The response of glutamate was delayed compared to Lac – The change in glutamate tended to decrease over time

Lin et al. (42)	10 adults	– 7 T – STEAM TE 15 ms – Midline visual cortex – 2 cm × 2 cm × 2 cm	– Visual stimulation included contrast-defined wedges, moving toward or away from the fixation cross and randomized – Two protocols: (1) 1 13.2 min block interspersed by rest epochs and (2) two 9.9 min blocks interspersed by rest epochs	– Increased glutamate (2 ± 1%) during single block vs rest – Increased glutamate (3 ± 1%) during the two blocks vs rest	

Schaller et al. (43)	10 adults	– 7 T – SPECIAL TE = 6 ms – Midline visual cortex – 2 cm × 2 cm × 2 cm	– Reversed black–gray checkerboard (9 Hz) – 2 blocks interspersed by rest epochs	– Increased glutamate (4 ± 1%) during stimulation vs rest	

Bednařík et al. (44)	12 adults	– 7 T – Semi-LASER TE = 26 ms – Midline visual cortex – 2 cm × 2 cm × 2 cm	– Red–black checkerboard (7.5 Hz) – 2 blocks interspersed by rest epochs	– Increased glutamate (~3%) during checkerboard vs rest

Apšvalka et al. (45)	19 young adults	– 3 T – PRESS TE = 105 ms – Left lateral occipital cortex – 2 cm × 2 cm × 2 cm	– Three different task blocks: novel stimuli and two repeated (6 unique vs 4 unique) stimulus presentations interspersed with rest blocks – Presentation of novel/repeated black-line drawings representing real world objects for 700 ms – 4 runs of 8 task blocks per run – Each run, 4 novel and 4 repeated blocks – Each block 36 s in duration	– Increased glutamate (~12%) during novel presentations compared to both rest and repeated presentations

**Motor task—motor and somatosensory cortex**					

Schaller et al. (35)	11 adults	– 7 T – SPECIAL TE = 12 ms – Left motor and somatosensory cortices – 1.7 cm× 2 cm × 1.7 cm	– Cued finger-to-thumb tapping task with both hands at a frequency of 3 Hz – 2 blocks interspersed by rest epochs	– Increased glutamate (2 ± 1%) during finger tapping vs rest

**Thermoregulation—anterior cingulate cortex (ACC) and insular cortex**					

Mullins et al. (47)	12 adults	– 4 T – STEAM TE = 20 ms – Bilateral ACC – 2 cm × 2 cm × 2 cm	– Frozen compress (0–4°C) or sham pain was applied to the base of the left foot – 8:32 min task epoch preceded by a rest block and followed by two 8:32 min rest periods	– Increased glutamate (9 ± 6%) during pain condition vs rest condition	

Gussew et al. (48)	6 adults	– 3 T – PRESS TE = 30 ms – Left anterior insular cortex – 2.5 cm × 1 cm × 1 cm	– Heat stimuli were applied to the inner skin area of the left forearm – 2 blocks interspersed by rest epochs	– Increased glutamate (18 ± 8%) during heat vs rest

**Executive functions—dorsolateral prefrontal cortex (dlPFC)**					

Woodcock et al. (40)	16 young adults	– 3 T – PRESS – TE = 23ms – left dlPFC – 1.5 × 2.0 × 1.5 cm^3^	– 2-back working memory task – 7 task blocks of 64 s each interspersed by 32 s rest epochs	– Increased glutamate (2.7%) during n-back vs fixation crosshair	– The control condition was a separate run fixating on a crosshair

Lynn et al. (87)	16 young adults	– 3 T – PRESS – TE = 23ms – left dlPFC – 1.5 × 2.0 × 1.5 cm^3^	– Four “non-task-active” conditions: relaxed eyes closed, passive visual fixation crosshair, visual flashing checkerboard, and a finger tapping task – Each task 3:28 min in duration	– Increased glutamate (4.7 and 3.2%) during flashing checkerboard and motor finger tapping conditions, respectively compared to visual fixation crosshair condition – Visual fixation crosshair and visual flashing checkerboard conditions produced the least variability in glutamate with CV’s under 5%, which were both significantly lower compared to the eyes closed condition with a mean CV = 6.7%	– Conditions were chosen because the left dlPFC is not the dominant brain region engaged in these tasks

**Learning and memory—hippocampus**					

Stanley et al. (36)		– 3 T – PRESS TE = 23 ms – Right anterior hippocampus – 1.7 cm × 3.0 cm × 1.2 cm	– Associative learning and memory task – Epochs of encoding (9 unique object–location pairs) and cued-retrieval (of those associated memoranda) and interspersed with rest epochs – 8 encoding-retrieval cycles were employed to allow learning to asymptote	– Increased glutamate (5.2 and 4.2%) during both encoding and retrieval, respectively – Applying a median split based on learning proficiency, fast learners showed increased during the early encoding trials, whereas slow learners showed increased glutamate in the later encoding trials	– Motor finger tapping task in response to a random visual stimulus was the control condition

**Cognitive control—ACC**					

Taylor et al. (66)	7 adults	– 7 T – STEAM TE = 10 ms – dACC – 2 cm × 2 cm × 2 cm	– STROOP task with 4 conditions – One block flanked by rest epochs	– Increased glutamate (2.6 ± 1.0%) during STROOP vs rest	– Significance based on one-tailed *t*-test

Taylor et al. (65)	16 controls; 16 major depressive disorder (MDD); 16 Schizo	– 7 T – STEAM TE = 10 ms – dACC – 2 cm × 2 cm × 2 cm	– STROOP task with four conditions – Two blocks interspersed with rest epochs	– Increased glutamate (3.2%) in controls during first STROOP vs rest – Decreased glutamate in MDD during second STROOP vs rest

**Visuospatial cognition—parietal and posterior cingulated cortices**					

Lindner et al. (68)	19 adults	– 3 T – PRESS TE = 32 ms – Right or left border of parietal/occipital cortices – 1.5 cm × 1.5 cm × 1.5 cm	– Visuospatial attention task – Button press in response to the tilt orientation of the grating that appeared on the side of the screen cued by an arrow – 3 conditions (ipsi, contra, and control) randomized – 3 blocks interspersed with rest epochs	– No trial condition effect on glutamate

In this review, we focus on ^1^H MRS findings pertaining to changes in glutamate with task in the context of ^1^H fMRS [for a review on ^1^H fMRS of GABA, see Duncan et al. (16)]. First, we describe the technological advancements in ^1^H MRS that made characterization of the glutamate temporal dynamics with a temporal resolution under a min possible. Second, we survey the findings from seminal ^1^H fMRS studies demonstrating task-related changes in glutamate (Table 1). Third, we discuss the significance of observing changes in glutamate from the perspective of neurovascular and neurometabolic processes and evaluate the implication of the findings for understanding behaviorally relevant neural output driven by shifts in the E/I balance. Finally, we evaluate the pros and cons of the ^1^H fMRS application in studying normal and impaired cognitive functions and outline the challenges ahead.

## Technical Advancements

The history of ^1^H fMRS application to neuroimaging is to a large extent similar to that of the BOLD-based fMRI. Early ^1^H fMRS studies conducted in the 1990s on 1.5 and 2.0 T MR systems demonstrated decreases in glucose and (transient) increases in lactate localized to the visual cortex during visual stimulation, and the findings were interpreted as reflecting a transient increase in non-oxidative glycolysis (17–21). However, the recent emergence of high-field MR systems including 3, 4, and 7 T (and higher), have dramatically rejuvenated the MRS field. The major advancement was the increase in the signal-to-noise ratio (S/N), which scales with the B_0_ field strength. The enhanced S/N at higher B_0_ field strengths can improve the spatial resolution of the localized single-voxel ^1^H MRS to ~2–4 cm^3^ as well as significantly increase the temporal resolution of the ^1^H MRS acquisition to under a minute. In addition, the chemical-shift but not the scalar J-coupling of spin systems (e.g., CH_2_, CH_3_, etc.) scales with the B_0_ field strength, which in turn significantly improves in the spectral resolution or delineation of the coupled spin systems between molecules such as glutamate and glutamine (22). These advancements improve the overall accuracy and precision of quantifying glutamate and other metabolites (23, 24), minimized the partial volume effects that impeded voxel placement precision in functionally relevant brain areas, and more importantly, enabled capturing real-time task-induced changes in the brain biochemistry within the time scale of epochs often used in task-based fMRI paradigms.

In addition to the advantages of conducting high B_0_ field ^1^H MRS, recent improvements in the acquisition technology enabled acquisition of highly reliable ^1^H MRS data with minimal spectral artifacts (25). These new developments include the incorporation of B_1_-insensitive adiabatic excitation and refocusing radio-frequency (RF) pulses (26) and customized phase- and amplitude-modulated RF pulses (27), which greatly improve the uniformity of the B_1_ field and edge profile of the defined MRS voxel. As a result, the ground was set for resurgent popularity of ^1^H MRS acquisition sequences such as the Localization by Adiabatic SElective Refocusing (LASER) (26), semi-LASER (28), and SPin ECho, full Intensity Acquired Localized (SPECIAL) (29). Adiabatic pulses are highly effective for outer volume suppression, which is a typical part of the acquisition sequence (30). Regarding the suppression of the water signal, the CHEmical Shift Selective (CHESS) RF pulses (31) has become common. However, optimized schemes using CHESS pulses such as the Variable Power and Optimized Relaxation delays (VAPOR) technique are robust and highly effective in suppressing the water signal and producing a cleaner spectral baseline (32).

Maximizing the homogeneity of the B_0_ magnetic field *via* shimming is critical for attaining optimal spectral resolution, especially in brain areas with extreme B_0_ susceptibility (e.g., the hippocampus or orbital frontal cortex). Techniques such as the Fast Automatic Shim Technique using Echo-planar Signal readouT for Mapping Along Projections (FASTESTMAP) (33) and its predecessor, FASTMAP (34), have brought significant improvement in the spectral quality, including increased S/N (35, 36). These acquisition sequences are readily available by most manufacturers on current MR systems and should be utilized [for review see Duarte et al. (37)].

Finally, reliable voxel placement across subjects and within subjects over time is an often-overlooked aspect of single-voxel ^1^H MRS acquisition protocols (25). Unreliable voxel placement adds error variance to the outcome measurements by increasing the variability of the partial volume effect. Recently introduced automated approaches have demonstrated significant improvements in consistency of voxels placement, between subjects, even in anatomical brain areas, in which partial voluming is difficult to avoid, such as the dorsolateral prefrontal cortex (dlPFC) (38, 39). For example, Woodcock et al. (40) reported an improvement from 68% voxel overlap with manual placement to 98% overlap using an automated approach. In all, these major technological advancements provide the necessary tools to fully exploit the characterization of the task-related temporal dynamics of glutamate and GABA with ^1^H fMRS, which is fueling the resurgence of *in vivo* ^1^H fMRS as a powerful tool for cognitive neuroscience and psychiatry research.

## Evidence of Task-Induced Glutamate Modulation

### Visual Stimuli—Visual Cortex

As in BOLD-based fMRI, the visual cortex is one of the most studied brain regions with ^1^H fMRS (Table 1). Studies of response to flashing checkerboard stimuli compared to a non-visual stimulation (i.e., a blank screen) have shown a consistent stimulus-bound increases of ~2–4% in steady-state glutamate levels (41–44). The magnitude of the average task-related increase in glutamate may be less consistent as it depends on task duration and cognitive processing demands. Shorter stimulus blocks were associated with a 3% increase in glutamate, compared to 2% for longer ones (42). With a temporal resolution of ~1 min, a delay in the increased stimulus-dependent modulation of glutamate was consistently observed, whereas smaller and earlier elevations in lactate were noted (41, 43, 44). The mechanism of these two temporal effects is not fully understood. Finally, sensitivity of glutamate levels to stimulus characteristics was illustrated by a ^1^H fMRS study that found an almost 12% increase within the left occipital cortex during passive viewing of novel pictures compared to a (pseudo-) rest control condition, but no change during repeated picture presentation (45).

### Motor Task—Motor and Somatosensory Cortex

To date, only a single ^1^H fMRS study, at 7 T, investigated neurochemical changes in the motor cortex during a motor task (35). As expected, a periodic cued finger-to-thumb tapping induced a significant (2%) glutamate increase in the motor/somatosensory cortices relative to a non-tapping “rest” condition (Table 1). In that study, the ^1^H fMRS voxel was co-localized with BOLD fMRI activation. Thus, task-related changes in glutamate can be detected in other functionally relevant cortical areas besides the visual cortex and can be used in investigating interesting research questions pertaining to neural activity during implicit vs explicit motor learning or periodic vs randomly cued stimuli (46).

### Thermoregulation and Pain Perception—Anterior Cingulate Cortex (ACC) and Insular Cortex

Motivated by the involvement of the ACC in thermal sensory responses, Mullins et al. (47) investigated glutamate response to a 10 min cold-pressor stimulation of the foot compared to the baseline rest without the cold exposure. They observed a substantial (9.3%) condition-related increase in glutamate within the ACC. With acute heat exposure, Gussew et al. (48) reported an even greater, 18%, glutamate increase in the anterior insular cortex. The manipulation involved acute 5 s cycles of heat exposure to the forearm compared to the no heat exposure condition. These findings lay the foundation of further investigation of the brain’s thermoregulatory system and its relationship to temperature perception with greater temporal resolution, made possible by current improvements in ^1^H MRS.

### Working Memory (WM)—dlPFC

The construct of WM refers to the ability to hold information in memory for a duration of a few seconds while manipulating this information “on-line” in order to carry out a complex task (49). In primates, the dlPFC has been proposed as the central neural substrate of WM (50). Neuroimaging studies using PET and fMRI have confirmed the importance of the dlPFC, but also have implicated additional brain regions, such as the inferior parietal lobule and cerebellum (51, 52). In a recent ^1^H fMRS study with a single-voxel placement in left dlPFC, a significant 2.7% increase in glutamate was observed during a standard 2-back WM task compared to a continuous visual crosshair fixation in healthy young adults (Table 1) (40). The elevation in dlPFC glutamate observed with a temporal resolution of 32 s is consistent with the engagement of that region in WM processing that has been revealed by task-based BOLD fMRI studies. However, increased glutamate was more pronounced during the first-half compared to the second-half of the 64 s block. This suggests a temporal variation in the dlPFC engagement during WM task. This temporal effect has not been reported in fMRI studies using the N-back WM paradigm and warrants further investigation to determine whether the disengagement over time is related to WM proficiency. In all, the observed temporal dynamics of WM-related modulation of dlPFC glutamate provides a solid basis for new means of evaluating the effects of cognitive intervention, pharmacological therapies, or manipulation of the physiological (e.g., stress-provoking) conditions.

### Learning and Memory—Hippocampus

Glutamate plays a key role in learning and memory *via* its activity in the frontal and hippocampal circuits. The hippocampus is particularly rich in glutamatergic neurons, and memory consolidation in the hippocampus depends on synaptic plasticity mediated by glutamatergic *N*-methyl-d-aspartate (NMDA) receptors (53, 54). In addition, firing rate of hippocampal neurons is associated with acquisition of new associative memories (55). Therefore, it is plausible that memory processing would be linked to increased modulation of hippocampal glutamate, presumably driven by increased activity at NMDA receptors. This hypothesis was tested by Stanley et al. (36). During performance of an associative learning task with object–location pairs, healthy adults displayed, as expected, unique temporal dynamics of glutamate modulation in the right hippocampus (Table 1). In this ^1^H fMRS application with a 54 s temporal resolution, the epochs of memory consolidation and retrieval were clearly differentiated by the temporal pattern of glutamate modulation. Moreover, the temporal dynamics of glutamate modulation were associated with learning proficiency: fast learners demonstrated up to 11% increase in glutamate during the early trials, whereas a significant but smaller and later increase of 8% was observed in slow learners. These results are in accord with the notion of altered glutamatergic neuroplasticity as the central mediator of learning and memory. The observed link between memory performance and glutamatergic system activity is particularly important given the proposed role of glutamatergic dysfunction as the core phenomenon in cognitive aging, age-related neurodegenerative disorders such as Alzheimer’s disease (AD), and severe psychiatric conditions such as schizophrenia. Structural changes in the hippocampus and its subfields, especially CA1, which is enriched in glutamatergic neurons, have been observed in the course of cognitive aging and AD (56–59). Although the mechanisms of these changes remain unclear, regional gray matter shrinkage observed on MRI is likely to reflect reduction of neuropil, to which dendritic arborization and dendritic spines contribute a significant volume fraction (60). Dendritic spine density is highly plastic and is driven by changes in Ca^2+^ flux modulated by glutamatergic activity (61). It is plausible to assume that impairment in glutamate modulation may eventually result in reduced dendritic plasticity and contribute to regional neuropil shrinkage. Therefore, impairment of task-related glutamatergic modulation may provide a very early marker for impending cognitive dysfunction and a valuable instrument of monitoring response to interventions that are aimed at mitigating the targeted cognitive declines.

### Cognitive Control—ACC

The ACC plays a key role in multiple higher cognitive processes including monitoring and evaluating conflict in information processing (62, 63). The Stroop task, which requires naming the color of displayed words when the name of the color matches the color of the displayed word (congruent trials) and when the color does not match the color of the displayed word (incongruent trials), is commonly used to assess conflict-monitoring engagement. BOLD fMRI studies using the Stroop task have consistently shown increased activation in the dorsal ACC related to trials of high conflict and with low top–down control (64). Based on this premise, Taylor et al. (65) investigated whether the Stroop task can induce a change in glutamate in the dorsal ACC of healthy adults using ^1^H fMRS at 7 T (Table 1). Compared to the rest condition, a 2.6% increase in glutamate was reported during the Stroop task, which included a mixture of congruent and incongruent conditions as well as trials with words only (no color) and color only (no words). However, differences in dorsal ACC glutamate modulation between trail conditions within the Stroop were not reported.

In another study using the similar Stroop task with ^1^H fMRS at 7 T, Taylor et al. (66) extended the investigation to individuals with major depressive disorder (MDD) and schizophrenia. The observation of increased glutamate level in the dorsal ACC during the Stroop task compared to rest was replicated in healthy adults. However, no significant change in glutamate was observed in individuals with schizophrenia, while participants with MDD demonstrated decreased glutamate in the dorsal ACC during the task compared to rest. The non-significant change in glutamate with task in the participants with schizophrenia appears consistent with decreased BOLD fMRI activation during Stroop in schizophrenia (67). Interestingly, the lower glutamate in the dorsal ACC during Stroop in MDD may reflect a shift in the E/I balance toward decreased excitability that is potentially driven by an increase in the inhibitory drive (see Figure 1 and below for further discussion).

### Visuospatial Cognition—Parietal and Posterior Cingulate Cortices

Tasks involving the visuospatial attention and memory system were recently investigated using ^1^H fMRS at 3 T (Table 1). In healthy individuals, a non-significant modulation of glutamate was observed in the parietal–occipital cortex during a visuospatial attention task compared to the control condition (68). In another study, no significant task-related glutamate modulation was observed in the parietal–posterior cingulate cortex of healthy adults, patients with AD and individuals with amnestic mild cognitive impairment who performed a face-name associative memory task compared to the rest control condition (69). In both studies, details on the variability of the glutamate measurements were omitted and, therefore, it remains unclear whether the method afforded detection of a task-related change in glutamate of the order of 10% or less. It may be possible that the selected tasks were not at the level of difficulty that produced significant variations in glutamate level or that dynamics of glutamate are inherently weaker in the examined locations compared to the hippocampus and prefrontal cortex. Also, the lack of specific behavioral constraints during the rest condition might have increased variability in glutamate within brain areas that show BOLD fMRI activation under rest. Therefore, rest, under these circumstances, may represent a nonspecific, yet, not truly task-free condition and thus a suboptimal choice as a control comparison. These remain among multiple questions to be addressed in the further development of the method.

## Biological Significance of Characterizing Glutamate Modulation

The observed dynamic changes in glutamate levels during perceptual, motor, and cognitive tasks may open a new window into neural bases of normal and abnormal cognition and behavior. To accomplish that goal, the apparent brain changes in this key neurotransmitter must be linked to cellular and molecular processes that occur in the brain.

Neural activity generated in response to physiological stimuli triggers changes in many complex neurovascular and neurometabolic processes, including increased cerebral blood flow, glycolysis (CMR_Glc_), and oxidative metabolism (CMR_O2_), as well as synthesis of neurotransmitters (4, 5, 70–72)—all of which depend on significant increase in energy consumption. The temporal and spatial characteristics of these processes are not fully understood (4). Most notably, there is a mismatch (i.e., ~44 vs ~30%, respectively) between glucose utilization (non-oxidative CMR_Glc_) and oxygen consumption (CMR_O2_) in response to physiological stimuli (73, 74). Fox et al. (75) were the first to report this mismatch, which sparked the interest and focus of early ^1^H fMRS studies from the 1990s, as noted above (17–19, 21). However, more recent high-field ^1^H fMRS studies provided compelling evidence that the mismatch of ΔCMR_Glc_ > ΔCMR_O2_ is short-lived. It is necessary only for facilitating the transition to a new metabolic steady state following the onset of a physiological stimulus. It is this transitional change that is believed to be reflected by the dynamic changes of glutamate observed on ^1^H fMRS (35, 44, 71).

This transition between metabolic steady states is primarily driven by oxidative metabolism (71) is consistent with recalibration of excitatory and inhibitory activity balance in local circuits, and establishing an E/I equilibrium that underpins a new functional state of the brain (Figure 1) (4, 6, 7). At the synaptic level, following the release of glutamate, excess of the neurotransmitter is taken up by surrounding astrocytes and is subsequently converted, predominantly to glutamine, with the help of glutamine synthetase. Glutamine is then released and taken up by the presynaptic neuron where it is converted into glutamate by mitochondrial glutaminase, to complete the glutamate–glutamine cycle (76). A near 1:1 relationship between neuronal glucose oxidation and the glutamate–glutamine cycling (70, 77, 78) implies that the metabolic and neurotransmitter pools of glutamate, as typically viewed in the ^1^H MRS literature (79, 80), are tightly coupled and hence, indistinguishable by ^1^H MRS (70). Moreover, in astrocytes, the oxidative pathway regulates the glutamate turnover (synthesis and degradation) and the high-energy phosphate, adenosine triphosphate, can be generated to supply the demand of increased synthesis without the need of glycolysis (81). This association between increased excitatory synaptic neurotransmission and increased synthesis of exogenous glutamate provides a cellular basis for meaningful interpretation of glutamate measures obtained from ^1^H fMRS.

Translating this relationship to the macro-circuit level implies that glutamate levels and changes therein that are observed in a single-voxel by ^1^H fMRS reflect the net cortical output driven by the excitation and inhibition balance on local circuits. The implication is that a net increase in synaptic excitability is reflected at the cortical (macro-circuit) level as a relative increase in glutamate, which is observed on the signal produced by ^1^H fMRS (Figure 1) (6, 7). Notably, an opposite shift in the E/I balance on local circuits increases the inhibitory drive and consequently, decreases the net excitability, which is reflected in a relatively lower glutamate level registered on ^1^H fMRS. The salient point of this interpretation is that ^1^H fMRS is not simply indicating an “ON” or “OFF” brain response to stimulation but can reflect a stimulus-induced change in glutamate that reflects new metabolic steady states driven by relative shifts in the E/I equilibrium (Figure 1). Because cellular glutamate changes are tightly linked to synaptic plasticity (82), the apparent glutamate alterations observed on a macro level are likely to reflect experience-related plasticity as well. The implications of using ^1^H fMRS as a proxy of cellular process that are unobservable *in vivo* are far reaching. Further development and refinement of the method bodes well for the fields, in which the role of glutamate in core phenomena of behavior, cognition, and psychopathology has been established through the use of animal models (83). Fulfillment of these promises, however, hinges on resolving several key issues in methodology and interpretation.

## The Pros and Cons of ^1^H fMRS

The key advantage of ^1^H fMRS over the staple of cognitive neuroscience, BOLD-based fMRI, is that task-related changes in glutamate can be traced directly to established metabolic processes, and are not mediated by neurovascular effects. This relative directness of the method bypasses neurovascular mediators that may be affected by significant alterations of the vascular system and impairment of its regulation. Moreover, ^1^H fMRS is a quantitative method that can measure not only the magnitude of change in glutamate but its basal “non-task-active” steady-state level, which is not the case for fMRI. This makes the method particularly suitable for studying the neural basis of cognitive declines in older adults and persons with age-related neurodegenerative disorders, in whom vascular risk factors are highly prevalent and cognitively relevant (56). This relative directness of ^1^H fMRS is a feature that may significantly advance the understanding of brain dynamics underlying normal and abnormal cognition. To fulfill this promise, several key issues need to be addressed.

One unresolved concern is that as several groups have pointed out, there is evidence of a BOLD T_2_* effect on the spectral peaks including glutamate during task compared to the control condition. This T_2_* contribution narrows spectral linewidths by about 0.2–0.3 Hz in the visual cortex at 4 T, 0.5 Hz in the visual cortex at 7 T, and 0.25 Hz in the motor cortex at 7 T on task-related spectra (35, 41, 43, 44, 84, 85). This BOLD-linked confound, however, is yet to be reported at 3 T. In theory, the spectral fitting method such as LCModel (86) should account for changes in the spectral linewidth without influencing the accuracy of the metabolite quantification. Nonetheless, Mangia et al. (85) reported a non-significant reduction in glutamate levels with increasing spectral linewidth, which potentially suggests a bias on LCModel fitting. As a result, applying a linedwidth broadening to spectra acquired during task to ensure linewidths are matched across all spectra has become a common practice as part of the post-processing for 7 T ^1^H fMRS studies (35, 41, 43, 44, 85).

The magnitude of task-related change in glutamate levels vary considerably across the extant reports (Table 1), from as low as 2% up to 18%, and the reasons for such variability are unclear. This wide range may reflect multiple methodological variations among studies including sample size, acquisition protocol, and the differential accuracy and precision between field strengths (e.g., 3 vs. 7 T). Also, the participants’ characteristics and properties of the task may play a role in adding variability to the measured magnitude of the observed change. In most extant studies, the comparison condition was either a pseudo-rest state (i.e., passive state with no specific instructions), routine motor activity, or visual fixation on a stimulus without specific task-related instructions. The purpose of the control condition is to assess a steady state level of glutamate to be contrasted with those that are associated with task activity. For example, in the ^1^H fMRS study of the hippocampus by Stanley et al. (36), the control condition paradigm included a cued finger to thumb tapping task due to its strong attention and motor processing, without any learning or memory components. Likewise, the dlPFC ^1^H fMRS study by Woodcock et al. (40) incorporated a visual fixation crosshair condition as the baseline control condition, again, to minimize any potential dlPFC engagement during the control condition. Moreover, Lynn et al. (87) demonstrated differences in steady-state levels of glutamate as well as variability of glutamate in the left dlPFC across different conditions where the primary functional specialization of the dlPFC was not associated to these conditions (e.g., relaxed with eyes closed, visual fixation crosshair, visual flashing checkerboard, and motor finger tapping). The visual fixation crosshair condition demonstrated the lowest and less variable glutamate over the acquisition period compared to the relaxed eyes closed condition. The latter is consistent with studies reporting greater variability in glutamate during rest epochs compared to task (44, 88). Also, the steady-state glutamate level was significantly higher during the visual flashing checkerboard compared to the visual fixation crosshair condition. This is surprising considering that the left dlPFC is not the primary brain area for visual stimuli but is involved in multiple cognitive operations including deployment and maintenance of attention (89–92). We surmise that substantial variability in glutamate levels over time occurs during conditions in which behavior is poorly constrained (e.g., pseudo-resting state), and that better-defined and constrained non-cognitive control tasks such as visual fixation or finger tapping, are a better choice for baseline condition for frontal areas of interest. This hypothesis merits further empirical testing.

To make an *in vivo* method truly useful in investigating task-related changes it is imperative to establish high reliability and temporal stability of task-related glutamate measures. No such evidence is currently available for ^1^H fMRS, and reliability studies are urgently needed.

Because cognitive activity occurs in a wide range of time windows and calls for multiple interacting brain circuits, not every task may be equally suitable for investigation with ^1^H fMRS. Investigation of task properties and relevant brain locations that maximize the validity of ^1^H fMRS findings is necessary for optimization of the ^1^H fMRS application to investigating complex cognitive and psychiatric phenomena. Of critical importance is leveraging ^1^H fMRS animal studies that can use more sensitive methods that are available for human studies and are, therefore, critically important for validation of the method (93–96). It is important, however, to apply these methods not only with precision and degree of invasive control that are available in animal models but also with parameters that are equivalent to those that are suitable for humans. Such *translational harmonization* of methods is critically important in the understanding of task-related glutamate changes observed in human subjects.

Finally, ^1^H fMRS is still a project in progress. The one aspect of the method that significantly improved over the years is the temporal resolution of acquiring the glutamate signal, which has been brought well under a minute (40). The advantage of high temporal resolution is the possibility of investigating temporal course of glutamate change within relatively short-lived stages of cognitive processing (96), and gauging the course of modulation within a task block (36, 40). However, it may take ~1–2 min for glutamate to reach its maximum level following stimulus onset (35, 41, 43). This may reflect the time needed for the synaptic reorganization process, shifting the E/I balance in the local circuits, and establishing the new steady state of glutamate. On the other hand, a relatively rapid change in glutamate within the dlPFC during the WM task has been reported. Glutamate surge was greater during the first half of each 64 s block than the second one (40). Thus, examining the patterns of glutamate modulation as a function of various time scales is as important as refining temporal resolution of the method.

## Conclusion

^1^H fMRS is an exciting and promising technique that can offer important insights into the neurochemicals underpinnings of cognition and their temporal dimensions. In this review, we summarize preliminary but compelling evidence demonstrating the ability of ^1^H fMRS to detect changes in glutamate during various perceptual, motor, and cognitive tasks. Moreover, the method can detect changes in glutamate modulation that are induced by manipulations that affect cognitive performance. It is highly plausible that these 2–18% task-related changes in glutamate reflect transitions to new metabolic steady states driven by relative shifts in the E/I equilibrium through synaptic plasticity. Within this conceptual framework, ^1^H fMRS provides a sensitive tool for investigating the neural basis of cognitive operations that are directly relevant to specific deficits in psychiatric disorders or neurodegenerative diseases associated with advanced age.

## Author Contributions

Both authors contributed to the writing of the manuscript.

## Conflict of Interest Statement

The authors declare that the research was conducted in the absence of any commercial or financial relationships that could be construed as a potential conflict of interest.
